# Age‐associated reduction of nuclear shape dynamics in excitatory neurons of the visual cortex

**DOI:** 10.1111/acel.13925

**Published:** 2023-07-21

**Authors:** Tanita Frey, Tomonari Murakami, Koichiro Maki, Takumi Kawaue, Naoki Tani, Ayaka Sugai, Naotaka Nakazawa, Kei‐ichiro Ishiguro, Taiji Adachi, Mineko Kengaku, Kenichi Ohki, Yukiko Gotoh, Yusuke Kishi

**Affiliations:** ^1^ Graduate School of Pharmaceutical Sciences The University of Tokyo Tokyo Japan; ^2^ New York University Grossman School of Medicine New York New York USA; ^3^ Graduate School of Medicine The University of Tokyo Tokyo Japan; ^4^ Institute for AI and Beyond, The University of Tokyo Tokyo Japan; ^5^ Institute for Life and Medical Sciences, Kyoto University Kyoto Japan; ^6^ Institute for Integrated Cell‐Material Sciences, Institute for Advanced Study, Kyoto University Kyoto Japan; ^7^ Liaison Laboratory Research Promotion Center IMEG, Kumamoto University Kumamoto Japan; ^8^ Institute for Quantitative Biosciences, The University of Tokyo Tokyo Japan; ^9^ Department of Energy and Materials, Faculty of Science and Engineering Kindai University Osaka Japan; ^10^ Department of Chromosome Biology Institute of Molecular Embryology and Genetics (IMEG), Kumamoto University Kumamoto Japan; ^11^ International Research Center for Neurointelligence (WPI‐IRCN), The University of Tokyo Tokyo Japan

**Keywords:** aging, neuronal activity, neurons, nuclear shape, nuclear stiffness, SUN1, visual cortex

## Abstract

Neurons decline in their functionality over time, and age‐related neuronal alterations are associated with phenotypes of neurodegenerative diseases. In nonneural tissues, an infolded nuclear shape has been proposed as a hallmark of aged cells and neurons with infolded nuclei have also been reported to be associated with neuronal activity. Here, we performed time‐lapse imaging in the visual cortex of *Nex‐Cre;SUN1‐GFP* mice. Nuclear infolding was observed within 10 min of stimulation in young nuclei, while the aged nuclei were already infolded pre‐stimulation and showed reduced dynamics of the morphology. In young nuclei, the depletion of the stimuli restored the nucleus to a spherical shape and reduced the dynamic behavior, suggesting that nuclear infolding is a reversible process. We also found the aged nucleus to be stiffer than the young one, further relating to the age‐associated loss of nuclear shape dynamics. We reveal temporal changes in the nuclear shape upon external stimulation and observe that these morphological dynamics decrease with age.

## Graphical abstract

In vivo time‐lapse imaging of excitatory neurons in the visual cortex of young mice reveals dynamic changes in the nuclear shape in response to visual stimulation. However, in aged mice, the dynamics of morphological changes in the neuronal nucleus are reduced. Neuronal nuclei also become infolded and stiffened during aging.

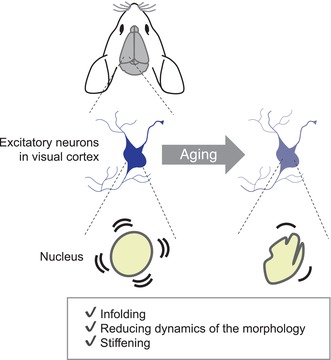

## 1 INTRODUCTION

Aging is associated with a decline in brain function, such as memory and cognition, and several neurodegenerative disorders, such as Alzheimer's disease, Huntington's disease (HD), and Parkinson's disease (PD) (Camandola & Mattson, 2017; Hof & Morrison, 2004; Hou et al., 2019). Identifying the mechanisms of brain aging is necessary for treating and preventing aging‐related conditions.

Among several hallmarks of the aging process, abnormalities of nuclear properties are common features of naturally aged and senescent cells in nonneural tissues (Heckenbach et al., 2022; Ragnauth et al., 2010; Scaffidi & Misteli, 2006). In neural tissues, the abnormal nuclear shape is observed in aging‐associated neurodegenerative diseases. In human patients with PD, neuronal nuclei are rather infolded, marked by their invaginated nuclear membrane, in contrast to the spherical shape observed in the hippocampus, frontal cortex, and substantia nigra (Liu et al., 2012; Shani et al., 2019). Furthermore, in PD‐model mice, with knockout or G2019S point mutation of the Lrrk2 kinase, the nuclei in the substantia nigra and dorsal thalamus showed more infolded nuclei (Chen et al., 2020; Shani et al., 2019). Similar infolded nuclear shapes in neurons were found in human patients with HD and the mouse model, overexpressing *HTT* genes with expanded CAG trinucleotide (Alcalá‐Vida et al., 2021; Gasset‐Rosa et al., 2017). These infolded nuclei in neurodegenerative diseases, such as PD and HD, showed defects in nuclear compartmentalization, and a more permeable nuclear membrane (Alcalá‐Vida et al., 2021; Shani et al., 2019). Nucleocytoplasmic transport of RNA was also impaired in neurons of the model mouse of HD (Gasset‐Rosa et al., 2017). These reports indicate the role of nuclear properties, such as nuclear shape, in the decline of neuronal function in neurodegenerative diseases; however, it is still unknown how the nuclear properties of neurons change over the time‐dependent natural aging process.

Nuclei in neurons also change their shape upon neuronal activity. Internal and external stimuli mediate neuronal activity and functional neuronal networks by inducing morphological changes in axons, dendrites, and synapses (Greer & Greenberg, 2008; Wong & Ghosh, 2002) and also by remodeling transcriptome during neuronal activity (Flavell & Greenberg, 2008). In the neuronal culture of the hippocampus and dorsal thalamus, neuronal activity induced via blocking GABA receptors using bicuculline increased the portion of infolded nuclei (Chen et al., 2020; Feurle et al., 2020; Wittmann et al., 2009). Also, the physiological stimulation by exposure to an enriched environment increased the amount of infolded nuclei in the CA1 of the hippocampus (Feurle et al., 2020). Nuclear infolding in neurons induced by neuronal activity is implicated in regulating gene transcription and nuclear compartmentalization. The neurons with infolded nuclei tended to have more phosphorylated histone H3 at serine 10, a histone modification for gene activation (Wittmann et al., 2009). Satb2, a neuronal transcription factor, is also involved in inducing the infolding of neuronal nuclei (Feurle et al., 2020), and it has been reported that multiple nuclear compartments separated by the invagination of nuclear membranes present different amounts of calcium signaling (Wittmann et al., 2009). An examination of the dynamics of the nuclear shape upon external stimuli in the physiological condition would further clarify our understanding of nuclear infolding and its role as would the age‐associated differences in the response of the nuclear shape upon external stimulation, which is currently completely unknown.

In this study, we analyzed the nuclear shape of excitatory neurons in the visual cortex of young and aged mice upon light stimulation after dark rearing. Previous reports have shown that excitatory neurons show nuclear infolding upon external stimuli (Feurle et al., 2020; Wittmann et al., 2009). We first confirmed the increased number of infolded nuclei in the stimulated visual cortex of young mice. To further reveal the dynamics of the infolding process in young mice, we performed time‐lapse imaging of the nuclear shape of excitatory neurons in the upper layer of the visual cortex of *Nex‐Cre;SUN1‐GFP* transgenic mice, whose nuclei in excitatory neurons were labeled with a GFP fused with SUN1 outer nuclear membrane protein (Goebbels et al., 2006; Mo et al., 2015). Over the course of light stimulation, nuclear shapes in the excitatory neurons of the visual cortex showed dynamic changes toward an infolded morphology, which was observed 10 min after visual cue exposure. After turning off the light stimulation, the nuclei's dynamics turned down, and the spherical shape reappeared, and was similar to conditions before the light stimulation, suggesting reversible changes in the nuclear morphology. We also examined nuclear shape in the aged visual cortex and found that excitatory neurons older than 2 years, even without visual stimulation, showed a higher portion of infolded nuclei than younger neurons. Time‐lapse imaging of aged mice revealed that the nuclei showed less frequent nuclear infolding upon visual stimulation, and atomic force microscopy (AFM) analysis revealed that aged neurons had stiffer nuclei. In our research, we first presented the temporal output of visual stimulation on the nuclear shape in the visual cortex of excitatory neurons and then the decline in the nuclear shape dynamics during the natural aging process.

## 2 MATERIALS AND METHODS

### 2.1 Animals

*ICR*, *C57BL/6J*, or *C57BL/6N* mice (CLEA Japan) were used for the study of wild‐type mice. Some of the aged mice were provided by the Foundation for Biomedical Research and Innovation at Kobe through the National BioResource Project of the MEXT, Japan. *Nex‐Cre* mice were provided by Klaus‐Armin Nave (Max Planck Institute), and *SUN1‐GFP* mice (#021039) were obtained from The Jackson Laboratory. The age of the mice is indicated in the figure legends. All mice were maintained in a temperature‐ and relative humidity‐controlled (23 ± 3°C and 50 ± 15%, respectively) environment with a normal 12‐h light and 12‐h dark cycle. Two to six mice were housed per sterile cage (Innocage, Innovive; or Micro BARRIER Systems) with chips (PALSOFT, Oriental Yeast; or PaperClean, SLC Japan), and irradiated food (CE‐2, CLEA Japan) and filtered water available ad libitum. All animals were maintained and studied according to protocols approved by the Animal Care and Use Committee of The University of Tokyo.

### 2.2 Immunohistochemistry

Immunohistochemistry was performed as previously described (Eto et al., 2020). Mice were injected intraperitoneally with pentobarbital solution (Nakalai Tesque) for anesthesia. Cold PBS was flushed via a 30G needle, followed by cold 4% paraformaldehyde (PFA) in phosphate‐buffered saline (PBS) on the left heart ventricle. After perfusion, the brains were removed and soaked overnight in 4% PFA on a rotator, and then they were incubated with 10%, 20%, and 30% sucrose in PBS and embedded with O.C.T. compound (Sakura Finetek Japan) at −80°C. Sixteen‐micrometer coronal sections of embedded brains were prepared by a cryostat. For staining, samples were exposed to antigen retrieval (10 min at 105°C in 1 × TRS [DAKO]). Then the samples were exposed to Tris‐buffered saline containing 0.1% Triton X‐100 and 3% bovine serum albumin (blocking buffer) for 2 h at room temperature, incubated first overnight at 4°C with primary antibodies (Table S2) in blocking buffer and then for 2 h at room temperature with Alexa Fluor‐conjugated secondary antibodies (1:1000, Thermo Fisher Scientific) in blocking buffer, and mounted in Mowiol (Calbiochem). All images were taken with Leica SP5, Zeiss LSM 880, or Olympus FV3000 as a z‐stack image to include the entire nucleus. For analysis of the images, ImageJ software (National Institute of Health) was used. Nuclei for which the entire image was taken were randomly selected from maximum projection images and analyzed for subjective counting of infolded or spherical nuclei, circularity, and nearest distance from the center of gravity. For subjective counting, infolded nuclei were defined by shaped or patterned signals inside the nucleus or by the outer shape being strongly invaginated. For circularity, area ratio, and nearest distance from the center of gravity, the GFP or Lamin B1 signals were binarized (Figure 2d). The inner nuclear shape (gray in Figure 2d) was used for the calculation of circularity. After determining the center of gravity on the basis of the outer nuclear shape, the distance from this center to the closest signal of GFP or Lamin B1 was normalized with the apparent radius.

### 2.3 Craniotomy and viral injection into the visual cortex

*Nex‐Cre;SUN1‐GFP* mice were prepared for in vivo imaging as described previously (Murakami et al., 2015; Ohki & Reid, 2014). Under stereotaxis conditions, which were performed under constant 2% isoflurane exposure, the skin over the skull was removed, and any muscle in proximity was cleanly cutoff to ensure a dry area on which the plate could be positioned and glued by the Super‐Bond dental cement (Sun Medical). A custom‐made metal head plate was glued on the skull, and the area of interest was located at the lateral region covering 2.5–4 mm from Lambda in the partial bone, the monocular region of visual cortex. The craniotomy was made over the entire visual cortex, and the dura (thin protective layer on the brain) was carefully removed. Five‐hundred nanoliters of the virus, AAV1‐Syn‐Flex‐NES‐jRGECOSa‐WPRE‐SV40 (Plasmid #100854, Addgene) (Dana et al., 2016), in saline was directly injected into the cortex at a speed of 1 nL/s by Nanoject III (Drummond), while covered by artificial CSF. Afterward, the window was closed with glass slips (5.5 and 4 mm glued onto each other) and use of clear glue and dental cement (ADFA, Shofu). The closed window was further covered for protection with dental silicone (Shofu). After approximately 2 weeks, the window was checked for its clarity and covered again with dental silicone. Five days prior to the recordings, the mice were put into a dark rearing box.

### 2.4 Two‐photon microscopy of the visual cortex of *Nex‐Cre;SUN1‐GFP* mice

Approximately 2 weeks after the craniotomy, the mice were exposed to darkness for 5 days in a dark rearing box and seated on a heat block during the entire time of recording. During anesthetized recordings, mice were sedated with chlorprothixene (0.3–0.8 mg/kg; Sigma‐Aldrich) and isoflurane (0.8%). The recordings were done with NIS‐Elements AR (Nikon) using a two‐photon microscope (Nikon A1MP) equipped with a mode‐locked Ti:sapphire laser (Mai Tai Deep See; Spectra Physics). The excitation light was focused with a 25× objective (Nikon Plan Apo, NA 1.10). GFP and jRGECO signals were excited at 960 nm, and the emission was filtered at 517–567 and 570–625 nm, respectively. A square region of the cortex (512 × 512 pixels, approximately 330 μm on a side) was imaged at two frames/min. Images were obtained from layer two of three (200–300 μm from the pia). Visual stimuli were generated using custom‐written programs in PsychoPy (Peirce, 2007). The stimulus presentation was synchronized with the frame acquisition of images using a counter board (NI USB‐6501; National Instruments). We positioned a 32‐inch LCD monitor 18 cm from the right eye of each mouse. Drifting rectangle strips of different angles were presented as waves in a randomized manner. For the conversion of the recordings, Matlab R2020b (version 4 9.9) was used. Note that we did not use the jRGECO signals for further analysis because of insufficient image quality. For analysis of the images, ImageJ software was used. Nuclei whose centers were taken in the z‐axis direction were randomly selected for analysis of area ratio.

### 2.5 Mechanical testing for isolated nuclei using AFM

Cell nuclei of excitatory neurons in the visual cortex of *Nex‐Cre;SUN1‐GFP* mice were attached to the Poly‐D‐Lysine (PDL)‐coated Petri dishes (World Precision Instrument, FD5040‐100) and subjected to AFM‐based mechanical testing (Maki et al., 2016). In this study, we utilized JPK BioAFM NanoWizard 3 (Bruker Nano GmbH), which was mounted on an epi‐fluorescence microscope (IX81; Evident). The spring constants of AFM cantilevers (qp‐BioAC CB2; Nanoworld AG) were calibrated based on the thermal noise method (Butt & Jaschke, 1995). Cell nuclei of excitatory neurons were fluorescently identified with GFP. AFM mechanical testing for the GFP‐positive nuclei was conducted with the following settings; the piezo displacement speed of 6 μm/s and the sampling rate of 4000 Hz. Based on the indentation force (*F*) versus depth curve obtained by mechanical testing, we estimated slope (nN/μm) by linear regression for the sample points within a force range (500 pN ≤ *F* ≤ 1000 pN), as previously described (Ichijo et al., 2022; Maki et al., 2015).

### Statistical analysis

Data are presented as means ± SD and were compared with two‐tailed Welch's *t* test or by analysis of variance (ANOVA) followed by Tukey's multiple comparison test. A *p* value of <0.05 was considered statistically significant.

## 3 RESULTS

### 3.1 Infolding of nuclear shape within excitatory neurons increases upon visual stimulation in the visual cortex

Previous studies have examined the changes in nuclear shape via different evoked stimulation systems (Chen et al., 2020; Feurle et al., 2020; Wittmann et al., 2009). To study the in vivo nuclear shape behavior in relation to neuronal activity upon physiological stimulation, we utilized the visual system because of its simplified manipulative approach via ocular enucleation and dark rearing. We performed enucleation of the left eye, and the mice were exposed to 5 days of dark rearing to reduce the neuronal activity in the visual cortex to a baseline (Lyckman et al., 2008). After 4 h of light exposure, we confirmed the upregulation of *Npas4*, which is a representative of immediate early genes (IEGs) induced by neuronal activity by reverse‐transcribed quantitative PCR (RT‐qPCR) analysis (Figure S1a,b).

**FIGURE 1:**
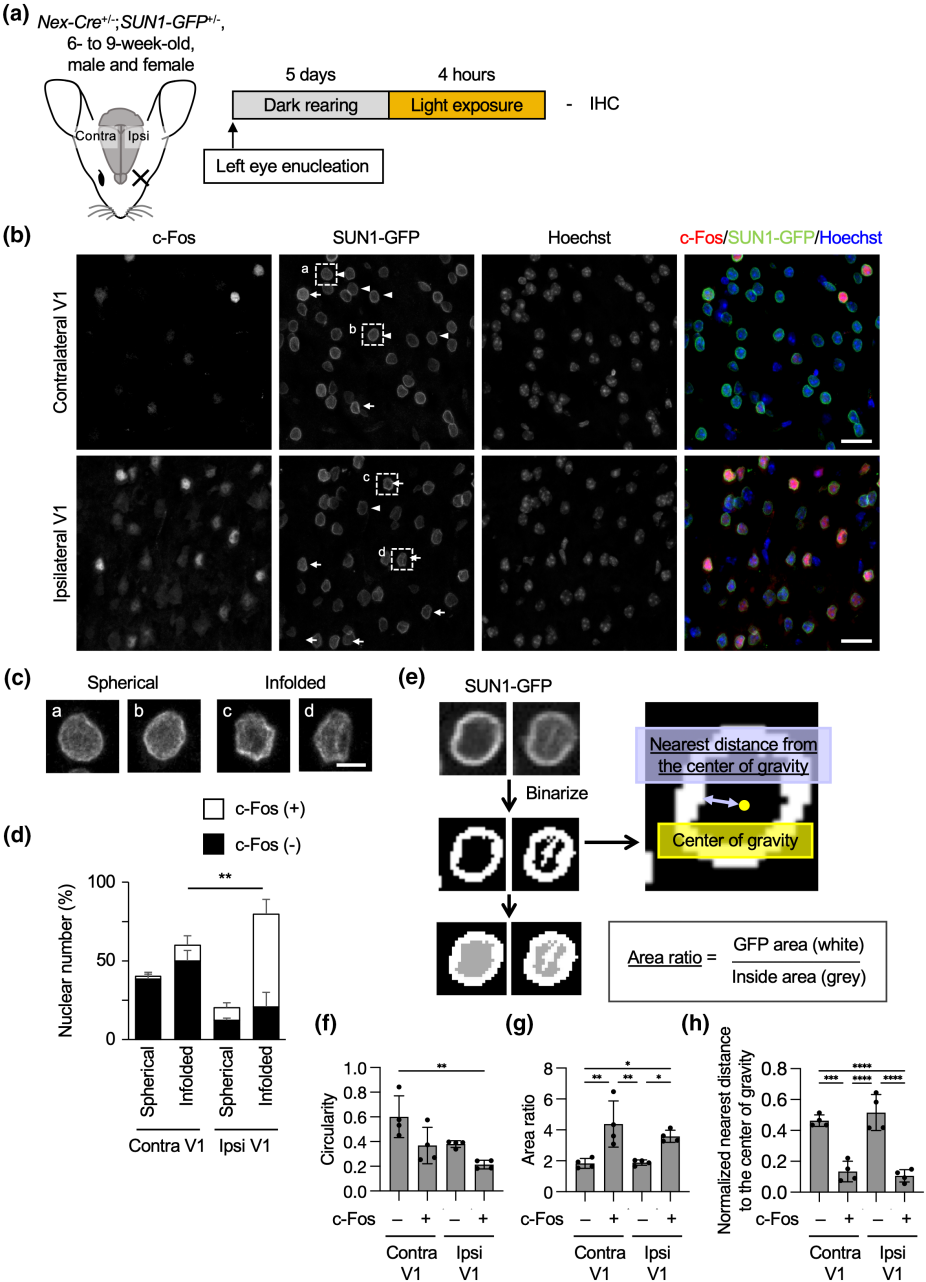
Nuclear infolding in the excitatory neurons in the primary visual cortex induced by visual stimulation. (a) Left eyes of 6‐ to 9‐week‐old *Nex‐Cre;SUN1‐GFP* mice were enucleated, and the mice were kept in a dark room for 5 days. They were stimulated by ambient light for 4 h and subjected to immunohistochemistry. (b) Coronal sections of the brain were stained with antibodies to c‐Fos and GFP. Nuclei were counterstained with Hoechst 33342. The images were obtained from layer 2/3 of the primary visual cortex. Arrows and arrowheads indicate infolded and spherical nuclei, respectively. Scale bars, 20 μm. (c) Higher magnification images of spherical and infolded nuclei indicated in (b). Scale bars, 5 μm. (d) Quantification of the proportion of c‐Fos–positive and spherical or infolded nuclear shapes among all GFP‐positive cells in all layers of the primary visual cortex. 1842 (contralateral) and 2926 (ipsilateral) nuclei from four independent experiments were analyzed. (e) Area ratio values were determined as the area of GFP signals (white) divided by the inside area (gray) after binarization of the nuclear image. The nearest distance from the center of gravity was determined as the distance from the center of gravity to the closest GFP signal. (f–h) Quantification of circularity (f) or area ratio (g) value of SUN1‐GFP signals or nearest distance from the center of gravity (h) in the ipsilateral and contralateral visual cortex. 232–233 (c‐Fos–negative, contralateral, circularity, and area ratio), 98 (c‐Fos–positive, contralateral, circularity and area ratio), 74 (c‐Fos–negative, ipsilateral, circularity and area ratio), 288 (c‐Fos–positive, ipsilateral, circularity, and area ratio), 158 (c‐Fos–negative, contralateral, and nearest distance), 64 (c‐Fos–positive, contralateral, and nearest distance), 107 (c‐Fos–negative, ipsilateral, and nearest distance), and 194 (c‐Fos–positive, ipsilateral, and nearest distance) nuclei from four independent experiments were analyzed. Data are mean ± SD *p* values between four groups were determined by one‐way ANOVA followed by Tukey's multiple comparison test. *p < 0.05, **p < 0.01, ***p < 0.001, ****p < 0.0001.

Using the visual manipulation, we aimed to examine the nuclear shape in excitatory neurons of the visual cortex in *Nex‐Cre;SUN1‐GFP* mice, which express the GFP‐fused SUN1 protein, a protein on the outer nuclear membrane, under the control of the *NeuroD6/Nex* gene locus (Goebbels et al., 2006; Mo et al., 2015). We used 6‐ to 9‐week‐old mice, in which the critical period of visual cortex development had finished, to rule out any effects due to the particular behavior of neurons during this period (Hensch, 2005; Hooks & Chen, 2007). The left eyes of the 6‐ to 9‐week‐old *Nex‐Cre;SUN1‐GFP* mice were enucleated, and the mice were kept in a dark room for 5 days and afterward were exposed to the light for 4 h (Figure 1a). The brain slices were immunostained with the antibodies for GFP and c‐Fos to evaluate neuronal activity (Figure 1b). The ipsilateral hemispheres of the removed left eye, which received light stimulation from the intact right eye, showed an increase in their c‐Fos expression in the visual cortex in comparison to the contralateral hemisphere, which did not receive light stimulation (Figure 1b,d). This supports the notion that our dark rearing of monocular mice and light stimulation appropriately manipulate neuronal activity, although the contralateral hemisphere contained c‐Fos–positive nuclei probably activated by spontaneous activity (Koukouli et al., 2022). The GFP staining pattern showed spherical (arrowheads) or infolded (arrows) nuclear shapes (Figure 1b,c), and their numbers were counted within the stimulated and deprived hemisphere of monocular enucleated mice (Figure 1d). In the contralateral hemisphere, 60% of total nuclei showed an infolded shape, which is consistent with previous reports analysing hippocampal neurons (Feurle et al., 2020; Wittmann et al., 2009). By contrast, in the ipsilateral hemisphere, the nuclear shape distribution showed a strong dominance of infolded nuclei (79%) in comparison to spherical nuclei (20%). Moreover, most of the infolded nuclei (74%) in the ipsilateral hemisphere were c‐Fos–positive, suggesting that physiological visual stimulation induces nuclear infolding in the visual cortex.

Since distinguishing nuclear shapes relied on the subjective observations of the SUN1‐GFP signal, we next evaluated the nuclear shape using three objective values: circularity, area ratio, and nearest distance from the center of gravity. Previous studies have evaluated nuclear infolding by reduction of circularity value (Alcalá‐Vida et al., 2021; Chen et al., 2020). Therefore, we first examined the circularity value of the inner area of the SUN1‐GFP signal (a gray area in Figure 1e) and found that the circularity of the c‐Fos–positive nuclei in the ipsilateral visual cortex was lower than the c‐Fos–negative nuclei in the contralateral one (Figure 1f). The circularity of c‐Fos–negative nuclei in the ipsilateral hemisphere was lower than that in the contralateral hemisphere, probably because c‐Fos–negative nuclei in the ipsilateral hemisphere might include neurons whose expression of c‐Fos transiently increased and already decreased before 4 h (Barros et al., 2015; Chowdhury & Caroni, 2018).

Along with the outer shape changes, we noticed that some nuclei presented the SUN1‐GFP signal inside the nuclei. Therefore, we also defined an area ratio value: the SUN1‐GFP signal area (a white area in Figure 1e) divided by the non–SUN1‐GFP occupied area (a gray area in Figure 1e) within the nucleus (Figure 1e). This means that a higher area ratio value represents a more infolded or non‐spherical nuclear shape. The area ratio of c‐Fos–positive nuclei was higher than that of c‐Fos–negative nuclei in both the contralateral and ipsilateral hemispheres (Figure 1g).

We further analyzed the distance between the center of gravity (a yellow point in Figure 1e) to the closest signal of GFP (a blue arrow in Figure 1e). The distance to the center of gravity of the c‐Fos–positive nuclei was shorter than that of the c‐Fos–negative nuclei in both the contralateral and ipsilateral cortex (Figure 1h). Taken together, these results suggest that the neuronal activity by light stimulation induced the shift of nuclear shapes toward an infolded shape within excitatory neurons of the visual cortex, and that this is a novel context of nuclear infolding of neurons induced by physiological stimulation.

### 3.2 The nuclear shape of excitatory neurons dynamically changes in response to visual stimulation in vivo

**FIGURE 2:**
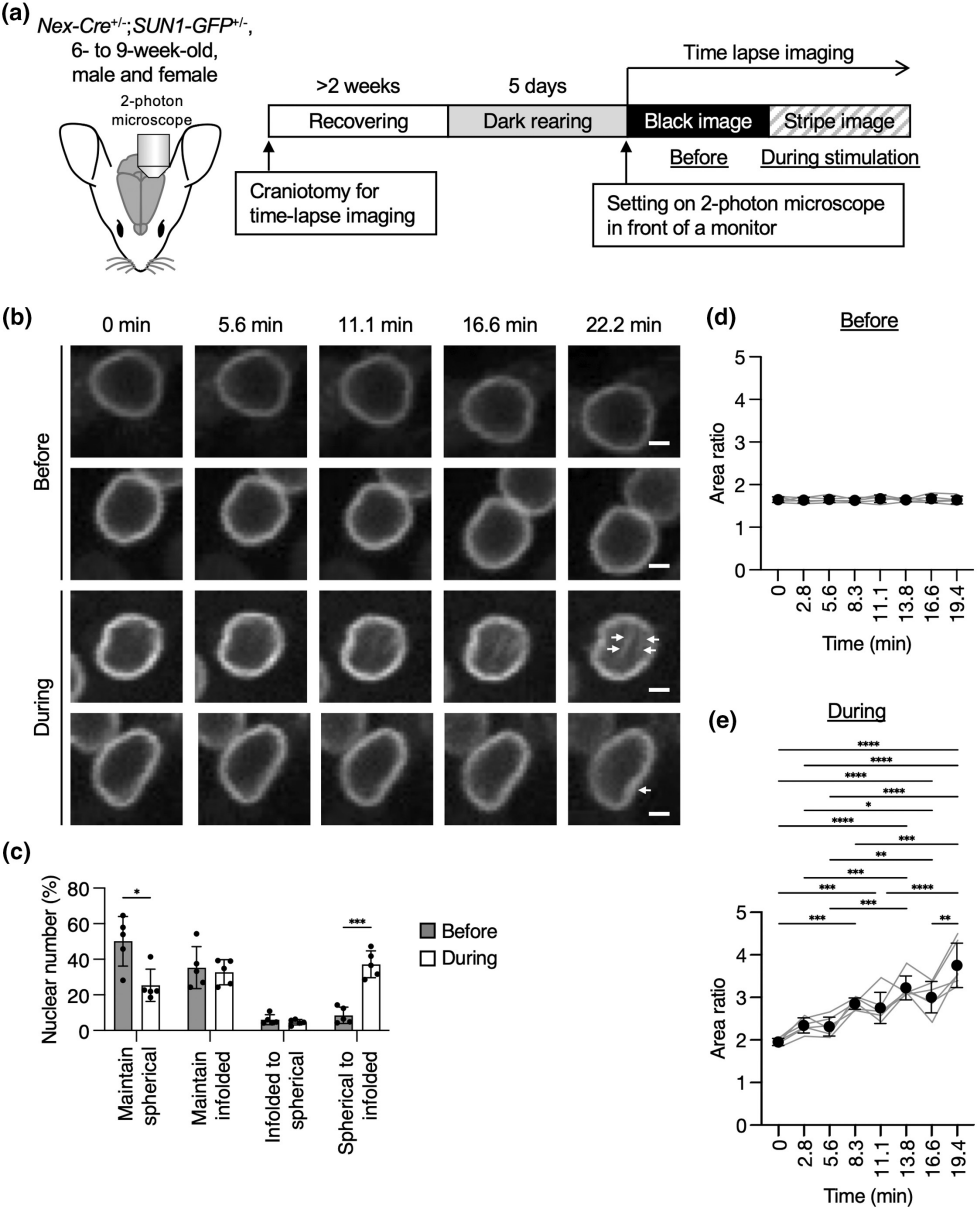
Time‐lapse imaging of the nuclear shape of the excitatory neurons in the primary visual cortex upon visual stimulation. (a) 6‐ to 9‐week‐old *Nex‐Cre;SUN1‐GFP* mice were subjected to craniotomy for time‐lapse imaging. After recovering for approximately 2 weeks and being kept in the darkroom for 5 days, the mice were set on the two‐photon microscopy in front of the monitor. The nuclear shape visualized by GFP in layer 2/3 was recorded at an interval of 30 seconds with the black image (“before”) and then with the striped image (“during”). (b) Representative images of GFP signals in “before” (upper two panels) and “during” (lower two panels) conditions. Arrows indicate an invaginated area at 22.2 min. Scale bars, 2 μm. (c) Quantification of the proportion of the nuclei showing the “maintained” or “changed” shape between spherical and infolded during the recording are shown. 322 nuclei in “before” and 305 nuclei in “during” from five independent experiments were analyzed. (d, e) Quantification of area ratio values at the indicated time in “before” (d) or “during” (e) recordings. 62 nuclei from five independent experiments (d) and 52 nuclei from five independent experiments (e) were analyzed. Data are mean ± SD. *p* values were determined by two‐tailed Welch's *t* test (c) or by one‐way ANOVA followed by Tukey's multiple comparison test (d, e). *p < 0.05, **p < 0.01, ***p < 0.001, ****p < 0.0001.

As nuclear infolding can be observed upon exposure to visual stimulation in excitatory neurons within the visual cortex, this raises the question of the infolding process and its temporal relationship to neuronal activity. Therefore, we performed live imaging of the upper layer of the visual cortex in *Nex‐Cre;SUN1‐GFP* mice in vivo with two‐photon microscopy during visual light exposure following the prior preparations of craniotomy on the visual cortex for time‐lapse imaging (Figure 2a). After the craniotomy, the mice recovered for more than 2 weeks and then underwent harvesting in a dark room for 5 days. Then, for the “before light stimulation” recordings, using low‐dose anesthesia, we placed the mice in front of a black monitor displaying a black image for approximately 1 h. We considered this recording as a direct control for the following stimulation conditions in the same individual. For visual stimulation, the mice were exposed to spatially randomized visual cues presented on the monitor, separated by 4 s of gray screen presentations. Imaging of SUN1‐GFP signals was displayed for approximately 20 min before the light stimulation showed a stable and spherical nuclear shape of excitatory neurons in the upper layer (Figure 2b; Video S1). However, the nuclear shape changed dynamically and became infolded with invagination, indicated by arrows in Figure 2b, during the light stimulation (Figure 2b; Video S2). We classified the nuclear shapes into changed or maintained and spherical or infolded before and during the exposure to light stimulation (Figure 2c). We found that the majority of the nuclei (50%) maintained their spherical shape before stimulation, while 37% of the nuclei during stimulation changed their nuclear shape from spherical to infolded.

For objective evaluation of nuclear shape, we analyzed the area ratio value defined in Figure 1e. Before light stimulation, the area ratio did not change for 20 min (Figure 2d). However, during light stimulation, the area ratio started to significantly increase around 10 min of exposure (Figure 2e). We also noted the significant variety of area ratio values between different mice after 15 min of exposure. This is likely a result of differences in sensory response between individuals (Lathe, 2004). Indeed, a previous report showed differences in Ca^2+^ levels upon visual stimulation in the primary and higher visual cortex between individuals (Murakami et al., 2017).

In summary, our time‐lapse imaging suggests that the nuclear shapes of excitatory neurons in the visual cortex require approximately 10 min to begin altering their shape upon visual stimulation. As immediate responses after stimulation for neurons, MAP kinases are activated within 5 min, and they expressed IEGs within 20 min (Tyssowski et al., 2018). Our results indicate that nuclear infolding is another immediate response of neurons upon external stimulation.

### 3.3 Dynamic behavior in nuclear shapes decreases after neuronal activity in excitatory neurons is inhibited

**FIGURE 3:**
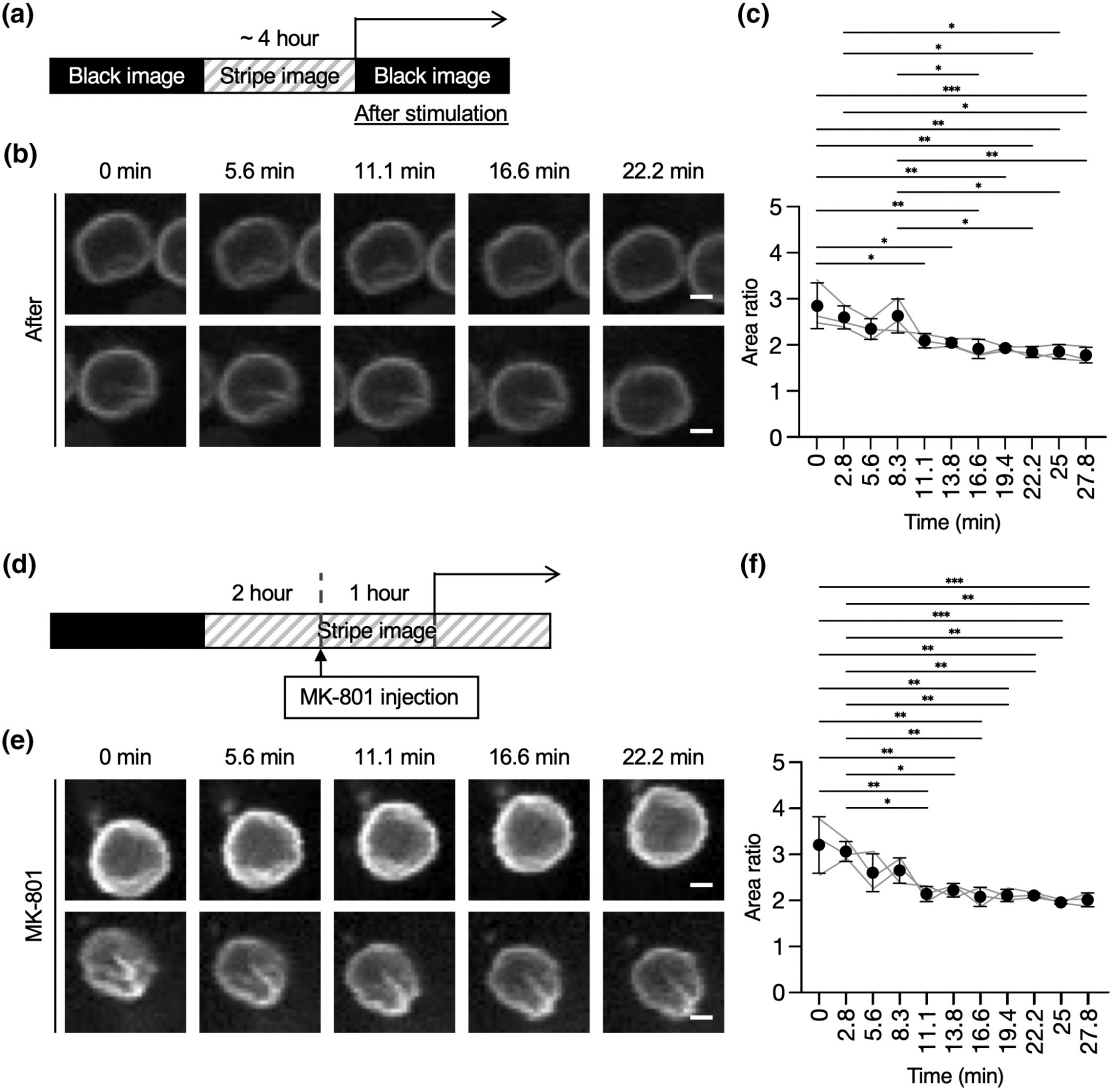
The behavior of the nuclear shape after inhibiting neuronal activity. (a) Four hours after visual stimulation as in Figure 2, the mouse was re‐exposed to the black monitor and the nuclear shape visualized by GFP was recorded. (b, c) Representative images of GFP signals (b) or quantification of area ratio values at the indicated time (c) in “after” conditions. Thirty‐seven nuclei from three independent experiments were analyzed. (d) Two hours after visual stimulation as in Figure 2, the visual cortex was injected with MK‐801. After 1 h, nuclear shape visualized by GFP was recorded. (e, f) Representative images of GFP signals (e) or quantification of area ratio values at the indicated time (f) in “MK‐801” conditions. Forty nuclei from three independent experiments were analyzed. Scale bars, 2 μm. Data are mean ± SD. *p* values were determined by one‐way ANOVA followed by Tukey's multiple comparison test. *p < 0.05, **p < 0.01, ***p < 0.001.

The strong relationship between neuronal activity and nuclear shape raises the question of what would happen to the nuclear shape once the neuronal activity is inhibited. First, we examined the changes in SUN1‐GFP patterns after turning off the visual stimulation by switching to the black image on the monitor (Figure 3a). During the first 10 min, the dynamic behavior of nuclear shapes gradually decreased and at 25 min became similar to the pre‐stimulation area ratio value (Figure 3b,c; Video S3), suggesting that the nuclear shape takes approximately 20 min to inhibit the dynamics observed during stimulation.

Additionally, we also examined another method by which to inhibit the effect of neuronal activity—pharmacological inhibition of calcium influx. MK‐801 is an NMDA receptor antagonist, and its administration reduces calcium influx and neuronal activity. To confirm that MK‐801 interferes with neuronal activity, the expression patterns of IEG in 6‐week‐old *C57BL6/J* wild‐type mice were analyzed. Following the 5 days of dark‐rearing treatment, the mice were exposed to light for 2 h and then injected with MK‐801 (Figure 3d). RT‐qPCR analysis of the visual cortex showed the weakened expression of *c‐Fos* in brains injected with MK‐801 compared to those injected with saline, confirming the effect of MK‐801 on the neuronal activity induced by the visual stimulation (Figure S1c). On this basis, we performed time‐lapse imaging to evaluate the effect of MK‐801 on the visual cortex of *Nex‐Cre;SUN1‐GFP* mice following 2 h of light exposure and examined the response of the nuclear shape upon the NMDA inhibitor application while presenting visual cues. We imaged the visual cortex approximately 1 h after injection and found that the nuclear shape had changed and the area ratio decreased despite the presence of light stimulation (Figure 3e,f; Video S4). These results suggest that NMDA activation and the calcium influx in excitatory neurons of the visual cortex are necessary for the dynamic changes in nuclear shape.

Taken together, these results indicate that the dynamic behavior of nuclear shapes induced by neuronal activity is a reversible event in an in vivo physiological system.

### 3.4 The nuclei in aged mice are more infolded compared to those in young mice

In addition to the neuronal activity‐induced nuclear infolding, previous studies have shown more infolded nuclei in human patients with PD and HD and model mice for PD and HD (Alcalá‐Vida et al., 2021; Chen et al., 2020; Gasset‐Rosa et al., 2017; Liu et al., 2012; Shani et al., 2019). As both PD and HD are neurodegenerative diseases associated with aging, we next examined the shape and dynamics of the nuclei in aged mice. We prepared 7‐week‐old and 133‐week‐old *C57BL6/J* wild‐type mice and performed visual stimulation as in Figure 1 (Figure S2a). Upon collecting the brains, their slices were immunostained with the antibodies for c‐Fos and Lamin B1, a component of the inner nuclear membrane (Figure S2b). The c‐Fos staining confirmed that appropriate stimulation of the cells in the ipsilateral visual cortex occurred in both young and aged brains, as did reduced stimulation in the contralateral visual cortex. In the young brain, the nuclear shape observed by Lamin B1 exhibited more infolded than spherical morphology in the ipsilateral brain compared to the contralateral brain (Figure S2b,c). However, the nuclear shapes in aged mice demonstrated an overall infolded appearance regardless of ipsilateral or contralateral condition (Figure S2b,c). Also, the infolded nuclear state in the contralateral brain of aged mice was evaluated by circularity, area ratio, and nearest distance from the center of gravity analyses (Figure S2d–f), and indicated nuclear shapes presenting a resting infolded state independent of any stimulation in the aged brain.

**FIGURE 4:**
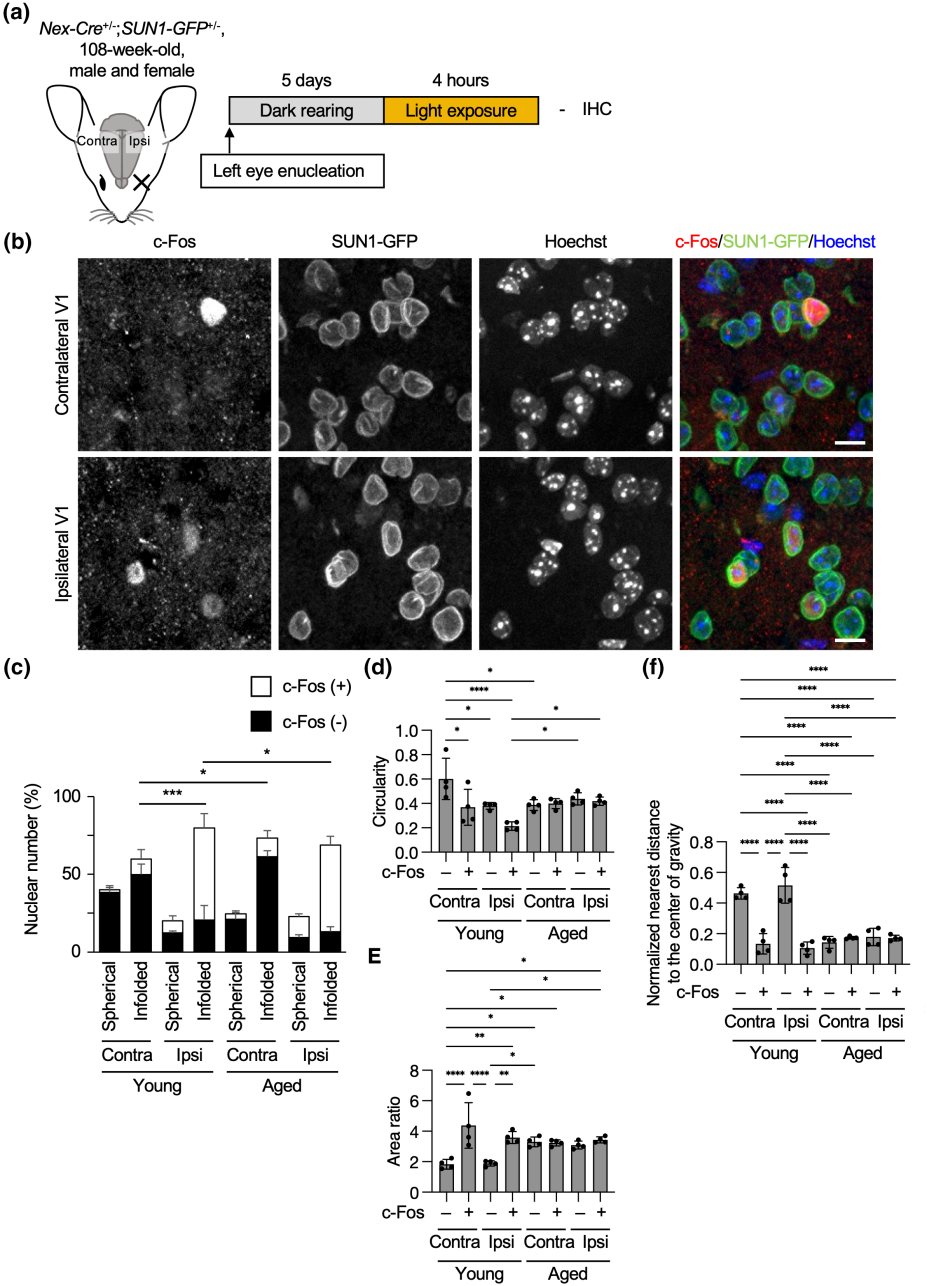
More infolded nuclei in the excitatory neurons in the aged primary visual cortex without visual stimulation. (a) One hundred and eight‐week‐old *Nex‐Cre;SUN1‐GFP* mice were analyzed as in Figure 1a. (b) Coronal sections of the brain were stained with the antibodies to c‐Fos and GFP. Nuclei were counterstained with Hoechst 33342. The images were obtained from layer 2/3 of the primary visual cortex. Scale bars, 10 μm. (c) Quantification of the proportion of c‐Fos–positive and spherical or infolded nuclear shapes in all layers of the primary visual cortex. 857 (contralateral) and 938 (ipsilateral) (d–f) Quantification of circularity (d), area ratio (e), or nearest distance from the center of gravity (f) in the ipsilateral and contralateral visual cortex of young and aged mice. 333 (contralateral) and 271 (ipsilateral) nuclei from four independent experiments were analyzed. All young data are the same in Figure 1. *p* values between eight groups were determined by one‐way ANOVA followed by Tukey's multiple comparison test. *p < 0.05, **p < 0.01, ***p < 0.001, ****p < 0.0001.

We next examined the changes in nuclear infolding during neuronal aging in a neuronal type‐specific manner. To compare the difference in nuclear infolding of young and aged excitatory neurons, we prepared 108‐week‐old *Nex‐Cre;SUN1‐GFP* mice and performed visual stimulation (Figure 4a). For the difference in neuronal response to visual stimulation during aging, the previous reports have shown that behavioral performance (visual acuity and contrast sensitivity) reduced with age (Lehmann et al., 2012) while the number of visual responsive neurons was comparable between young and aged animals (Mendelson & Wells, 2002). With our method, we could not observe a significant difference in c‐Fos–positive excitatory neurons between young and aged ipsilateral hemispheres (Figure 4c; 67% in young and 77% in aged, *p* = 0.21 by one‐way ANOVA followed by Tukey's multiple comparison test). GFP staining revealed more infolded nuclei in the aged contralateral hemisphere compared to younger hemispheres and no difference between contralateral and ipsilateral hemispheres in the aged brain (Figure 4b,c). The parameters of circularity, area ratio, and nearest distance from the center of gravity also showed more infolded shape in the aged brain without visual stimulation and no differences between the brains with and without visual stimulation (Figure 4d–f). However, when analyzing parvalbumin‐positive interneurons in young and aged wild‐type mice, we could not find consistent differences in circularity, area ratio, and nearest distance from the center of gravity between young and aged and between the contralateral and ipsilateral hemispheres (Figure S3a–d).

The immunohistochemistry results suggest that brain aging induces nuclear infolding dependent on neuron type. The nuclear infolding resembles the prior observed nuclear shapes in PD and HD brains (Alcalá‐Vida et al., 2021; Chen et al., 2020; Gasset‐Rosa et al., 2017; Liu et al., 2012; Shani et al., 2019), leading to the assumption that functional decline of the brain may be associated with infolded shapes of neuronal nuclei.

### 3.5 The nuclei in the aged brain have reduced nuclear shape dynamics compared to young nuclei

**FIGURE 5:**
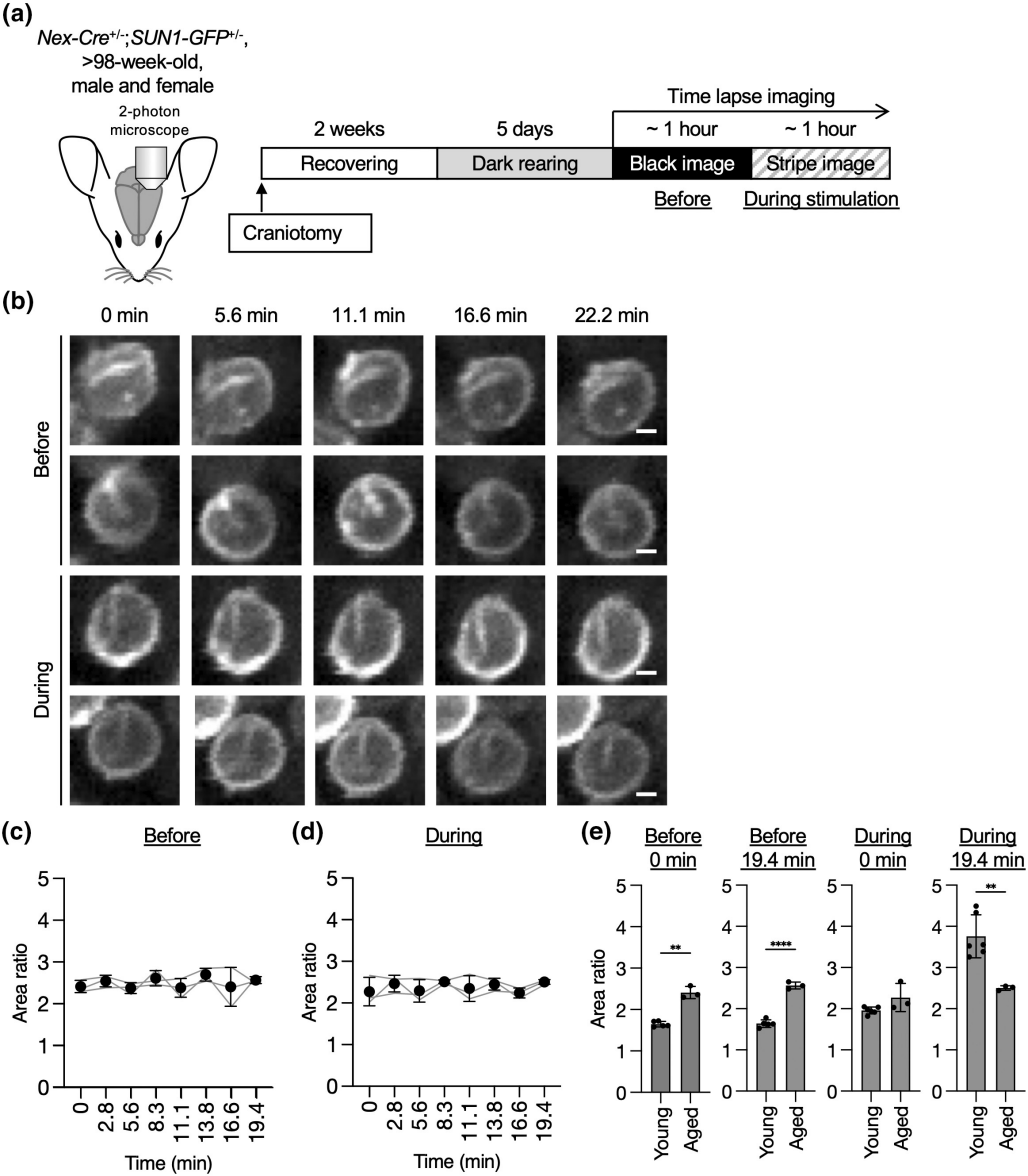
Less dynamic behavior of the nuclear shape in the aged brain. (a) *Nex‐Cre;SUN1‐GFP* mice older than 98 weeks old were analyzed as in Figure 2a. (b) Representative images of GFP signals in “before” (upper two panels) and “during” (lower two panels) conditions. Scale bars, 2 μm. (c, d) Quantification of area ratio values at indicated time in “before” (c) or “during” (d) recording. Thirty‐eight nuclei (c) and 43 nuclei (d) from three independent experiments were analyzed. (e) Comparison between area ratio values of the young in Figure 2d,e and of the aged in (c) and (d). Data are mean ± SD. *p* values were determined by two‐tailed Welch's *t* test. **p < 0.01, ****p < 0.0001.

To reveal the temporal dynamics of aged nuclei, we performed time‐lapse imaging of nuclear shape in upper layer neurons using *Nex‐Cre;SUN1‐GFP* mice that were more than 2 years old, as in Figure 2 (Figure 5a). Similar to the immunohistochemistry data (Figure 4b), we found that more nuclei presented an infolded shape and the area ratio value was significantly higher in aged mice compared to young mice even before stimulation (Figure 5b,c,e; Video S5). Additionally, the area ratio value was stable throughout imaging, and at 19.4 min, the value became significantly lower than that in the young brain (Figure 5b,d,e; Video S6). The lack of nuclear shape alteration in excitatory neurons toward visual stimulation in aged mice suggests that the dynamics of nuclei morphology reduce with age.

**FIGURE 6:**
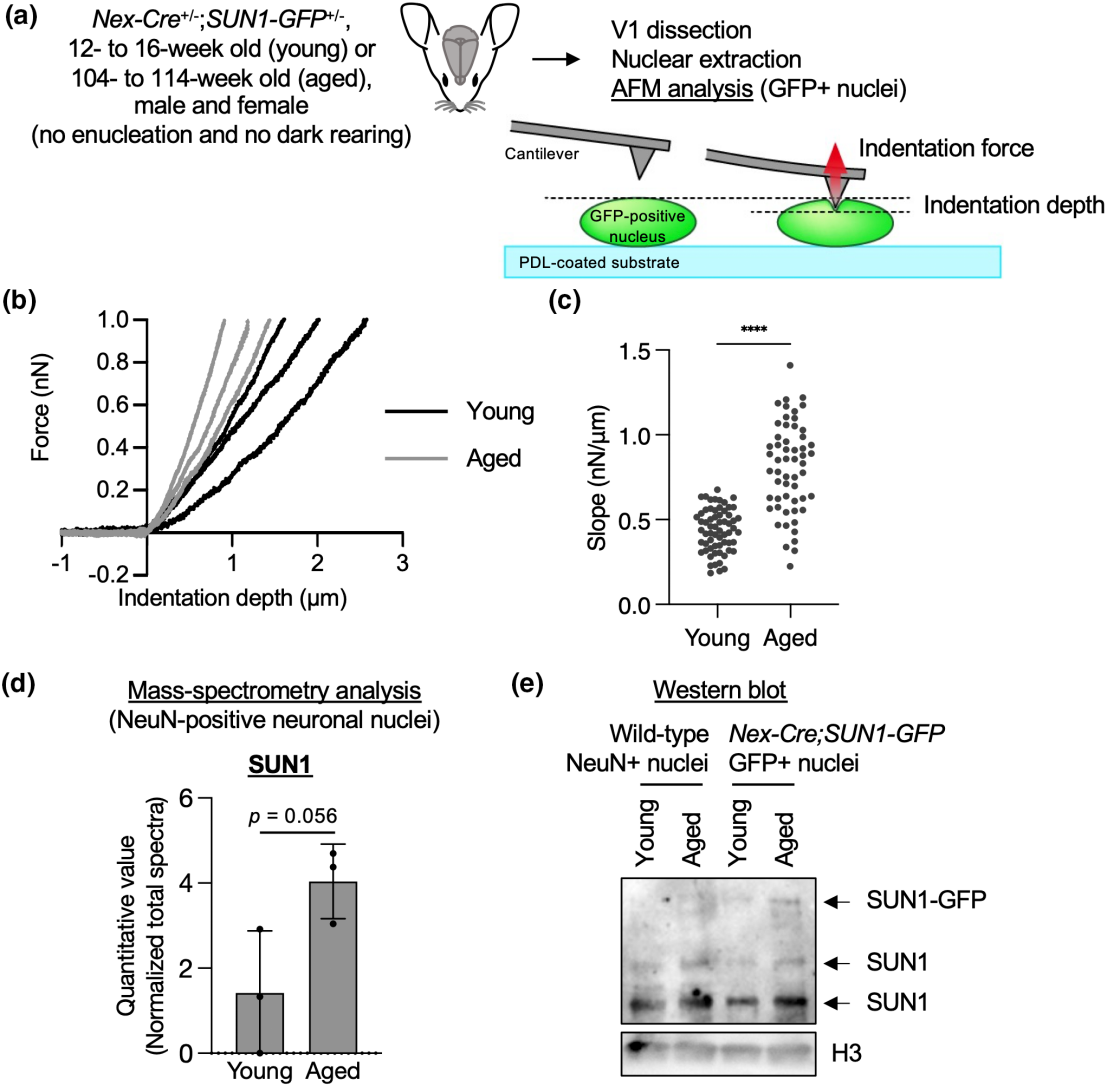
Stiffer nuclei of the excitatory neurons in the aged visual cortex. (a) Nuclei were isolated from the visual cortex of 12‐ to 16‐week‐old (young) or 104‐ to 114‐week‐old (aged) *Nex‐Cre;SUN1‐GFP* mice, and GFP‐positive nuclei were analyzed by AFM. The experimental scheme for AFM analysis is also shown. (b) Representative curves between indentation force and depth. Three curves each for young and aged nuclei are shown. (c) Beeswarm plots for the slope values in indentation force versus depth curve for nuclei. 63 young nuclei and 58 aged nuclei from three independent experiments were analyzed. *p* values were determined by two‐tailed Welch's *t*‐test. (d) NeuN‐positive nuclei isolated from the whole cortex of 7–10‐week‐old (young) or 108–119‐week‐old (aged) wild‐type mice by FACS were analyzed by LC–MS/MS. Data are mean ± SD from three independent experiments. *p* values were determined by Student's *t* test. (e) NeuN‐positive nuclei isolated from the whole cortex of 7‐ to 10‐week‐old (young) or 108‐ to 119‐week‐old (aged) wild‐type mice and GFP‐positive nuclei isolated from the whole cortex of 11‐week‐old (young) or 104‐week‐old (aged) *Nex‐Cre;SUN1‐GFP* mice by FACS were analyzed by western blot. A representative image from three independent experiments is shown. As previously reported, multiple bands were detected for endogenous SUN1 protein (Göb et al., 2011; Moujaber et al., 2017).

We next asked, why did the aged neurons show less frequent nuclear infolding events upon visual stimulation than young ones? Previous studies have shown that the nuclei of fibroblasts from patients with Hutchinson–Gilford progeria syndrome (HGPS), a premature aging disorder, were infolded and stiffer than those of the control cell line (Csoka et al., 2004; Eriksson et al., 2003; Goldman et al., 2004; Verstraeten et al., 2008), suggesting an association between nuclear infolding and stiffness. In addition, nuclear stiffness changes during development and under conditions of cellular stress (Nava et al., 2020; Pajerowski et al., 2007). Therefore, we assumed that stiffened nuclei would exhibit less frequent infolding upon aging when visually stimulated, and measured the stiffness of the isolated nuclei by AFM (Newberg et al., 2020). Nuclei were isolated from the visual cortex of 12‐ to 16‐week‐old or 104‐ to 114‐week‐old *Nex‐Cre;SUN1‐GFP* mice harvested under conditions without enucleation and dark rearing, using the Percoll density gradient centrifugation method (Bundo et al., 2016). Only GFP‐positive nuclei were subjected to AFM analysis, confirmed under an epi‐fluorescent microscope (Figure 6a). The nuclear stiffness was estimated from the relationship between the force exerted by the AFM cantilever and the indentation depth of the nucleus (Figure 6a). As a result, slope values in the indentation force versus depth curve, representing stiffness, within a force range (500 pN ≤ F ≤ 1000 pN) were significantly larger in aged nuclei (Figure 6b,c). This result revealed for the first time that aged nuclei were stiffer than young nuclei during the natural aging process. Therefore, the stiffening of the nuclei may cause resistance to deformation and the less frequent infolding events observed in aged neurons upon visual stimulation.

We next questioned the mechanisms by which the neuronal nuclei stiffened during aging using proteomics analysis. Neuronal nuclei labeled with an antibody for NeuN, a neuronal nuclear marker, were isolated from young and aged cortices from wild‐type mice by a fluorescent‐activated cell sorter (FACS) and subjected to mass spectrometry analysis (Table S3). Among identified proteins, we found that the amount of SUN1 protein increased in aged nuclei compared to younger nuclei (Figure 6d). Previous reports have shown that knockdown of SUN1 and SUN2 reduced nuclear stiffness (Guilluy et al., 2014; Newberg et al., 2020), suggesting the role of SUN1 in maintaining stiffness. Western blot analysis confirmed the increase of SUN1 protein in aged NeuN‐positive nuclei (Figure 6e). We were also concerned about the effect of ectopic expression of SUN1‐GFP protein in excitatory neurons to analyze stiffness in Figure 6c. Therefore, we compared the expression level of endogenous SUN1 and ectopic SUN1‐GFP proteins by western blot (Figure 6e). The SUN1‐GFP protein level was relatively lower than that of the endogenous SUN1 protein, and both SUN1‐GFP and endogenous SUN1 proteins increased in the aged brain. This indicates that the increase in SUN1 protein is a potential mechanism for nuclear stiffening during neuronal aging.

## 4 DISCUSSION

Nuclear infolding in neurons upon neuronal stimulation or neurodegenerative diseases has been reported in previous papers (Alcalá‐Vida et al., 2021; Chen et al., 2020; Feurle et al., 2020; Gasset‐Rosa et al., 2017; Liu et al., 2012; Shani et al., 2019; Wittmann et al., 2009), but its regulatory mechanism and significance have not been fully understood. In this study, we found that an in vivo physiological stimulation through light stimulation of the visual cortex induced nuclear infolding in excitatory neurons, similar to exposure to a novel environment for the hippocampal neurons (Feurle et al., 2020). Furthermore, time‐lapse imaging of the nuclear shape of excitatory neurons in the upper layer showed the dynamic behavior of the nucleus during light stimulation. As indicated in a previous study, immunocytochemistry showed that inhibition of neuronal activity with tetrodotoxin (TTX) in a hippocampal neuronal culture decreases the infolded nuclei (Wittmann et al., 2009). Our time‐lapse imaging of inhibition induced by light stimulation or MK‐801 injection also revealed that nuclear infolding upon stimulation is a reversible process.

Treatment with MK‐801 reduced the dynamic behavior, similar to the previous study using hippocampal slice culture (Wittmann et al., 2009). This suggests that calcium influx mediated by the NMDA receptor is upstream of nuclear infolding. In our imaging analysis, it took approximately 10 min of light stimulation for the dynamic behavior of the nucleus to begin. A previous study using hippocampal neuronal culture has shown the involvement of MAP kinase signaling in this process as MEK inhibitors, such as PD98059 and U0126, inhibited neuronal activity‐dependent nuclear infolding (Wittmann et al., 2009). Upon KCl stimulation, MAP kinase, such as Erk, becomes phosphorylated and activated within 5 min (Tyssowski et al., 2018). This is consistent with the idea that dynamic behavior and infolding of a neuronal nucleus are mediated by MAP kinase.

External stimuli induce the transcription of several genes in neurons and play an important role in the functional changes of neurons, such as neurite and synapse formation (Flavell & Greenberg, 2008; Greer & Greenberg, 2008). Among these induced genes, a gene set called IEGs or rapid primary responsive genes, including *c‐Fos* and *Arc*, increased their expression levels within 20 min of visual stimulation and decreased after turning off the stimulation (Tyssowski et al., 2018). This time course and reversibility are similar to the induction of nuclear infolding observed in the time‐lapse imaging of this study. Considering that the components of the inner nuclear membrane, such as Lamin, are involved in gene transcription through regulating chromatin structure, the changes in the nuclear shape may contribute to the regulation of gene transcription. This is further supported by the previous work studying the role of the interaction between Satb2, a neuronal transcription factor, and Lemd2, a component of the inner nuclear membrane, in nuclear infolding upon neuronal activity (Feurle et al., 2020). How then does nuclear infolding contribute to gene induction? One possibility is that nuclear infolding may allow rapid interactions between IEG loci and their regulatory elements. A previous Hi‐C experiment using hippocampal neurons after kainic acid injection has shown the chromatin interactions at the gene loci of responsive genes, such as *c‐Fos* and *BDNF*, within 1 h (Fernandez‐Albert et al., 2019). By contrast, the interaction between the nuclear membrane and the responsive gene loci, such as *Egr3* and *BDNF*, visualized by fluorescence in situ hybridization, has not been altered after bicuculline treatment in hippocampal neuronal culture (Noguchi et al., 2021). Therefore, the nuclear membrane may be required to be infolded in order for the regulatory element to interact with the gene loci while still bound to the nuclear membrane.

In the aged visual cortex of mice older than 2 years, the nuclei of excitatory neurons—but not the parvalbumin‐positive interneuron—generally demonstrated a higher portion of infolded nuclei than the young visual cortex in non‐stimulated conditions. In the previous study, the invagination of the nuclei of spiny projection neurons in the striatum of wild‐type mice did not increase in aged mice (2 years of age) compared to young mice (2 months of age) (Chen et al., 2020), also suggesting that age‐associated nuclear infolding is specific to neuron type. The neuronal nuclei in PD‐model mice were also bigger and more infolded than the nuclei of the controls (Chen et al., 2020). However, the area of the nuclei in the young and aged visual cortex was a similar size (Figure S4), further indicating neuronal type‐specific changes in nuclear shape during aging or neurological disease.

Then, what is the cause for the higher population of infolded nuclei in aged excitatory neurons? Long‐term stimulation (40 h) of hippocampal neurons by bicuculline induces a more stable infolded nuclear shape after TTX treatment compared to short‐term stimulation (1 h) (Wittmann et al., 2009). This suggests that more stimulation induces the fixed nuclear‐infolded shape. Aged excitatory neurons have naturally undergone repeated neuronal activity exposure and this may cause the irreversible infolded nuclear shape and the dysfunction of neuronal nuclei. In addition, the stiffer nuclei of aged neurons, possibly mediated by the increase of SUN1 protein, can contribute to the irreversible nuclear shape. Consistently, our time‐lapse imaging of nuclei of aged excitatory neurons revealed a decrease in dynamic behavior compared to those of young ones. The relationship between repetitive neuronal activity and nuclear alterations in frequency of morphological change, stiffness, and SUN1 expression level is unknown; however, their additional or synergistic contribution may induce nuclear infolding in the aged brain. Furthermore, elucidation of the similarities and differences between neuronal activity‐dependent and age‐dependent changes in nuclear shape requires further study (e.g., the profiling of genomic regions associated with the nuclear envelope).

In the present study, we compared only 2‐month‐old and 2‐year‐old mice and it is unknown whether neuronal nuclei become infolded at a certain time point between or gradually over time. It would be useful for future studies to analyze the time course of infolding of neuronal nuclei and speculate on the significance of the functional decline of aging neurons. However, the more infolded nuclear shape in the aged visual cortex in this study is reminiscent of the nuclear shape in PD and HD (Alcalá‐Vida et al., 2021; Gasset‐Rosa et al., 2017; Liu et al., 2012; Shani et al., 2019). Since both PD and HD are age‐associated neurodegenerative disorders, our results indicate that an increase in infolded nuclei is associated with the decline of neuronal functions. Previous studies have shown defects in the compartmentalization of infolded nuclei (Alcalá‐Vida et al., 2021; Gasset‐Rosa et al., 2017; Shani et al., 2019) and suggest that leakiness or abnormal nucleocytoplasmic transport of mRNA and proteins cause a functional decline of aged neurons. Indeed, previous studies revealed leaky nuclear membranes of aged brain cells and defects of protein import into the nucleus in aged human fibroblast (D'Angelo et al., 2009; Pujol et al., 2002). In another study, micropipette aspiration of the nuclear membrane of embryonic stem cells (ESCs), differentiated neural progenitor cells, and tissue fibroblast revealed a more deformable nuclear membrane of ESCs compared to differentiated cells (Pajerowski et al., 2007). ESCs have more plasticity for lineage choice and gene transcription (Meshorer & Misteli, 2006). Therefore, it is supposed that deformable nuclear membrane contributes to plasticity. Younger neurons also have more plasticity and are more adaptable to functional changes (Berardi et al., 2000; Katz & Crowley, 2002). Therefore, the less dynamic and stiffer nucleus in aged neurons may be related to their decrease in plasticity through regulating the events induced by neuronal activity, such as induction of IEGs.

## AUTHOR CONTRIBUTIONS

Tanita Frey and Yusuke Kishi designed the study and wrote the manuscript. Tanita Frey, Tomonari Murakami, Koichiro Maki, Takumi Kawaue, Naotaka Nakazawa, Ayaka Sugai, and Yusuke Kishi performed the experiments and analyzed the data. Tomonari Murakami and Kenichi Ohki assisted with time‐lapse imaging. Taiji Adachi and Mineko Kengaku assisted with AFM analysis. Naoki Tani and Kei‐ichiro Ishiguro assisted with mass spectrometry analysis. Yukiko Gotoh and Yusuke Kishi supervised the study. All authors contributed to the revision and approved the final version of the manuscript.

## CONFLICT OF INTEREST STATEMENT

The authors declare no competing interests.

## Supporting information


Data S1.

## Data Availability

The mass spectrometry data have been deposited in jPOST under the following accession numbers: PXD041560 for ProteomeXchange and JPST002125 for jPOST. Time‐lapse data have been deposited in SSBD under doi.org/10.24631/ssbd.repos.2023.06.302. All of the data in this study are available from the corresponding author upon reasonable request.
