# Supplementary material for: Age‐associated reduction of nuclear shape dynamics in excitatory neurons of the visual cortex
Source: Aging Cell. 2023 Jul 21;22(9):e13925. doi: 10.1111/acel.13925 (PMC10497821; doi:10.1111/acel.13925)
Supplement: Supplementary file 1 — Data S1. [file ACEL-22-e13925-s001.zip › acel13925-sup-0005-Supinfo.docx]

**Supplementary Information**

**Supplementary methods**

**Enucleation and dark rearing in mice for neuronal activity manipulation.**

The eye removal was performed under 2% isoflurane. The mouse was placed on a little pedestal. A tweezer clipped the optical nerve behind the eye, and then the eye was removed. The wound was glued via tissue adhesive application from Vetbond. After the left eye removal and the mouse’s recovery, they were exposed to 5 days of darkness within a box covered by a dark sheet to obtain the neuronal activity of the visual cortex to its lowest baseline.

**RT-qPCR analysis**

The visual cortex was dissected with a stainless steel 0.5 mm sagittal brain matrix for rodents. The brain was first cut at 1.5 mm and 3.5 mm from the midline. Then, the visual cortex was located over one-third of the posterior part of the hippocampus. The dissected visual cortex was put into RNAiso plus (Takara-Bio), and total RNA was precipitated. The amounts of obtained RNA were measured via NanoDrop One (Thermo Fisher Scientific), and 0.5 μg of the RNA was subjected to reverse transcription with the use of ReverTra Ace qPCR RT Master Mix with gDNA Remover (Toyobo). The resultant cDNA was subjected to real-time PCR analysis in a LightCycler 480 or LC96 instrument (Roche) with Thunderbird SYBR qPCR mix (Toyobo) or QuantiNova SYBR green PCR kit (Qiagen). The amount of each target mRNA was normalized by that of *GAPDH* mRNA. Primer sequences are provided in Supplementary Table 1.

**Microinjection of inhibitor**

The skin was opened and removed to insert a pinhole with a 30G needle onto the visual cortex (the caudal region around 4 mm from Lambda in the partial bone) in the mice under 2% isoflurane anesthesia in stereotaxis fixation. Five-hundred nanoliters of 100 mM MK-801 in saline was injected at a speed of 1 nL/s into the ipsilateral hemisphere by the Nanoject III. The right visual cortex, as a control, received a saline injection. The skin was stitched closed after injection.

**Nuclear isolation**

Nuclear isolation was performed as previously described (Bundo et al. 2016). The visual cortices of young and aged mice harvested under standard conditions (no enucleation and no dark rearing) were isolated as in RT-qPCR analysis and homogenized using a syringe with 23G and 27G needles, sequentially, in 500 μL of 54% Percoll in homogenizing buffer (50 mM Tris-HCl pH 7.4, 25 mM KCl, 5 mM MgCl_2_, and 250 mM sucrose) on ice. The solution was mixed with 10% NP-40 (final concentration, 0.1%), left on ice for 15 minutes, and mixed with 500 μL of homogenizing buffer. Then, Percoll gradient was prepared in the following order: 100 μL of 35% Percoll in homogenizing buffer on the bottom layer, 200 μL 31% Percoll in homogenizing buffer, and 1 mL of homogenate (27% Percoll) on the top layer. The tube was centrifuged at 20,000 x g for 10 minutes at 4°C. After removing the debris on the top layer, nuclei on the bottom layer were transferred into another tube.

**FACS**

For wild-type mice, isolated nuclei were incubated with Alexa 488–conjugated anti-NeuN antibody in 0.2% BSA/PBS for 10 min at 4°C, washed, and subjected to FACS with the FACSAria or FACSMelody instrument (Becton Dickinson). For *Nex-Cre;SUN1-GFP* mice, isolated nuclei were directly subjected to FACS.

**Mass spectrometry**

Mass spectrometry was performed as previously described (Tani et al. 2022). Isolated nuclei from the cortex of wild-type mice were resuspended with sample buffer (Nakalai Tesque, 09499-14), boiled for 30 min at 95°C, run on 4–12% NuPAGE (Thermo Fisher Scientific, NP0321) by 10 mm from the well, and stained with SimplyBlue (Thermo Fisher Scientific) for in-gel digestion. Excised gels containing nuclear proteins were cut into approximately 1 mm pieces, reduced with DTT (Thermo Fisher Scientific), and digested with trypsin and Lysyl endopeptidase (Promega) with 40 mM ammonium bicarbonate (pH 8.0) overnight at 37°C. The digested peptides were analyzed on an Advance UHPLC system (Bioscience, ABRME1ichrom) connected to a Q Exactive mass spectrometer (Thermo Fisher), and Xcalibur (Thermo Fisher Scientific) was used to process the raw mass spectrum. The raw LC-MS/MS data were analyzed with an NCBI non-redundant protein/translated nucleotide database restricted to *Mus musculus* using Proteome Discoverer version 1.4 (Thermo Fisher Scientific) with the Mascot search engine version 2.5 (Matrix Science). *p* values for each protein were determined with Scaffold 5 software. The raw LC-MS/MS data were deposited on ProteomeXchange with the accession number PXD041560 and on jPOST with the accession number JPST002125. The full list of LC-MS/MS data is shown in Supplementary Table 3.

**Western blot**

NeuN-positive or GFP-positive nuclei isolated from the cortex from wild-type or *Nex-Cre;SUN1-GFP* mice, respectively, were resuspended with sample buffer (Nakalai Tesque, 09499-14), boiled for 30 min at 95°C, and fractionated by SDS-PAGE on a 4–20% gel (Bio-Rad, 4561096). The separated proteins were transferred to a polyvinylidene difluoride membrane (Bio-Rad, 1704156) using TransBlot Turbo (Bio-Rad). The membrane was washed with PBS (Nakalai Tesque, 27575-31), incubated with blocking buffer (Toyobo, NYPBR01) for 30 min at room temperature, and incubated with the primary antibodies listed in Supplementary Table 2 in Can Get Signal Solution 1 (Toyobo) overnight at 4°C. Then the membrane was washed with PBS containing 0.2% Tween-20 and incubated with HRP-conjugated secondary antibodies (Cytiva, NA931V for mouse and NA9340V for rabbit) in Can Get Signal Solution 2 (Toyobo) for 30 min at room temperature. After washing with PBS-T, the membrane was processed for detection of peroxidase activity with chemiluminescence reagents (Bio-Rad, 170-5061) and iBright CL1000 (Thermo Fisher Scientific).

**Supplementary figure legends**

**Supplementary figure 1. Expression changes of an IEG upon visual stimulation after enucleation, dark rearing, light exposure, and MK-801 injection.**

**(A)** Left eyes of 6- to 9-week-old *ICR* wild-type mice were enucleated, and the mice were kept in a dark room for 5 days. They were stimulated by room light for 4 hours and subjected to reverse transcription and quantitative polymerase chain reaction (RT-qPCR). **(B)** RT-qPCR analysis of relative *Npas4* mRNA abundance (normalized by the amount of *Gapdh* mRNA) in the primary visual cortex was performed. **(C)** RT-qPCR analysis of relative *c-Fos* mRNA abundance (normalized by the amount of *Gapdh* mRNA) in the primary visual cortex after injection of saline or MK-801 was performed. Data are means ± s.d., averaged values for four mice. *p* values were determined by two-tailed Welch’s t-test. **p* < 0.05, ***p* < 0.01.

**Supplementary figure 2. More infolded nuclei in the aged cells in the primary visual cortex without visual stimulation.**

**(A)** 7- or 133-week-old *C57BL6/J* wild-type mice were analyzed as in Fig. 1A. **(B)** Coronal sections of the brain were stained with the antibodies to c-Fos and Lamin B1. Nuclei were counterstained with Hoechst 33342. The images were obtained from layer 2/3 of the primary visual cortex. Scale bars, 10 μm. **(C)** Quantification of the proportion of c-Fos–positive and spherical or infolded nuclear shape in all layers of the primary visual cortex. 1,200 (young, contralateral), 966 (young, ipsilateral), 1,096 (aged, contralateral), and 1,176 (aged, ipsilateral) nuclei from three independent experiments were analyzed. (**D–F**) Quantification of circularity (D) or area ratio (E) value of Lamin B1 signals or nearest distance from the center of gravity (F) in the ipsilateral and contralateral visual cortex of young and aged mice. 202 (circularity, young, contralateral), 184 (circularity, young, ipsilateral), 143 (circularity, aged, contralateral), 147 (circularity, aged, ipsilateral), 179 (area ratio, young, contralateral), 173 (area ratio, young, ipsilateral), 126 (area ratio, aged, contralateral), 41 (area ratio, aged, ipsilateral), 189 (nearest distance, young, contralateral), 167 (nearest distance, young, ipsilateral), 139 (nearest distance, aged, contralateral), and 150 (nearest distance, aged, ipsilateral) nuclei from three independent experiments were analyzed. *p* values were determined by one-way ANOVA followed by Tukey’s multiple comparison test. **p* < 0.05, ***p* < 0.01, ****p* < 0.001.

**Supplementary figure 3. Nuclear shape of parvalbumin-positive inhibitory neurons in the young and aged primary visual cortex with or without visual stimulation.**

**(A)** Coronal sections of the brain prepared as in Supplementary fig. 2 were stained with the antibodies to c-Fos, parvalbumin, and Lamin B1. Nuclei were counterstained with Hoechst 33342. The images were obtained from layer 2/3 of the primary visual cortex. Arrows indicate parvalbumin-positive cells. Scale bars, 10 μm. (**B–D**) Quantification of circularity (B) or area ratio (C) value of Lamin B1 signals or nearest distance from the center of gravity (D) in the ipsilateral and contralateral visual cortex of young and aged mice. 147 (young, contralateral), 161 (young, ipsilateral), 149 (aged, contralateral), and 176 (aged, ipsilateral) nuclei from three independent experiments were analyzed. *p* values were determined by one-way ANOVA followed by Tukey’s multiple comparison test. **p* < 0.05.

**Supplementary Video 1**

The nuclear shape of excitatory neurons in the visual cortex of 6- to 9-week-old *Nex-Cre;SUN1-GFP* mouse before stimulation. The nuclei shown in Fig. 2 are indicated as yellow arrows. This video is played at 120× speed.

**Supplementary Video 2**

The nuclear shape of excitatory neurons in the visual cortex of 6- to 9-week-old *Nex-Cre;SUN1-GFP* mouse during stimulation. The nuclei shown in Fig. 2 are indicated as yellow arrows. This video is played at 120× speed.

**Supplementary Video 3**

The nuclear shape of excitatory neurons in the visual cortex of 6- to 9-week-old *Nex-Cre;SUN1-GFP* mouse after stimulation. The nuclei shown in Fig. 3 are indicated as yellow arrows. This video is played at 120× speed.

**Supplementary Video 4**

The nuclear shape of excitatory neurons in the visual cortex of 6- to 9-week-old *Nex-Cre;SUN1-GFP* mouse after MK-801 injection. The nucleus shown in Fig. 3 was indicated as yellow arrow. This video is played at 120× speed.

**Supplementary Video 5**

The nuclear shape of excitatory neurons in the visual cortex of *Nex-Cre;SUN1-GFP* mice older than 98 weeks before stimulation. This video is played at 120× speed.

**Supplementary Video 6**

The nuclear shape of excitatory neurons in the visual cortex of *Nex-Cre;SUN1-GFP* mice older than 98 weeks during stimulation. The nuclei shown in Fig. 5 are indicated as yellow arrows. This video is played at 120× speed.

**Supplementary Table 1. Primer sequences for RT-qPCR**

| Gene | Forward primer | Reverse primer |
| --- | --- | --- |
| *GAPDH* | atgaatacggctacagcaacagg | ctcttgctcagtgtccttgctg |
| *Npas4* | ctgtccttgtagtgaactggtta | acaccaagtacagtgacttt |
| *c-Fos* | accctttgatgacttcttgt | gtctgctgcatagaaggaac |

**Supplementary Table 2. Antibodies for immunohistochemistry, FACS, and western blot**

| Antigen | Manufacturer | Serial Number | Concentration |
| --- | --- | --- | --- |
| GFP | Abcam | ab13970 | 1:2000 |
| c-Fos | Santa Cruz | sc-271243 | 1:100 |
| Lamin B1 | Abcam | ab16048 | 1:250 |
| Parvalbumin | Swant | PVG-213 | 1:2000 |
| NeuN-Alexa 488 | Merck | MAB377X | 1:400 |
| SUN1 | Novus | NBP2-59943 | 1:500 |
| Histone H3 | Abcam | ab1972 | 1:500 |

**Supplementary Table 3. Proteomics analysis for neuronal nuclei from the young and aged cortex**
