# Supplementary figures and images for: Age‐associated reduction of nuclear shape dynamics in excitatory neurons of the visual cortex
Source: Aging Cell. 2023 Jul 21;22(9):e13925. doi: 10.1111/acel.13925 (PMC10497821; doi:10.1111/acel.13925)

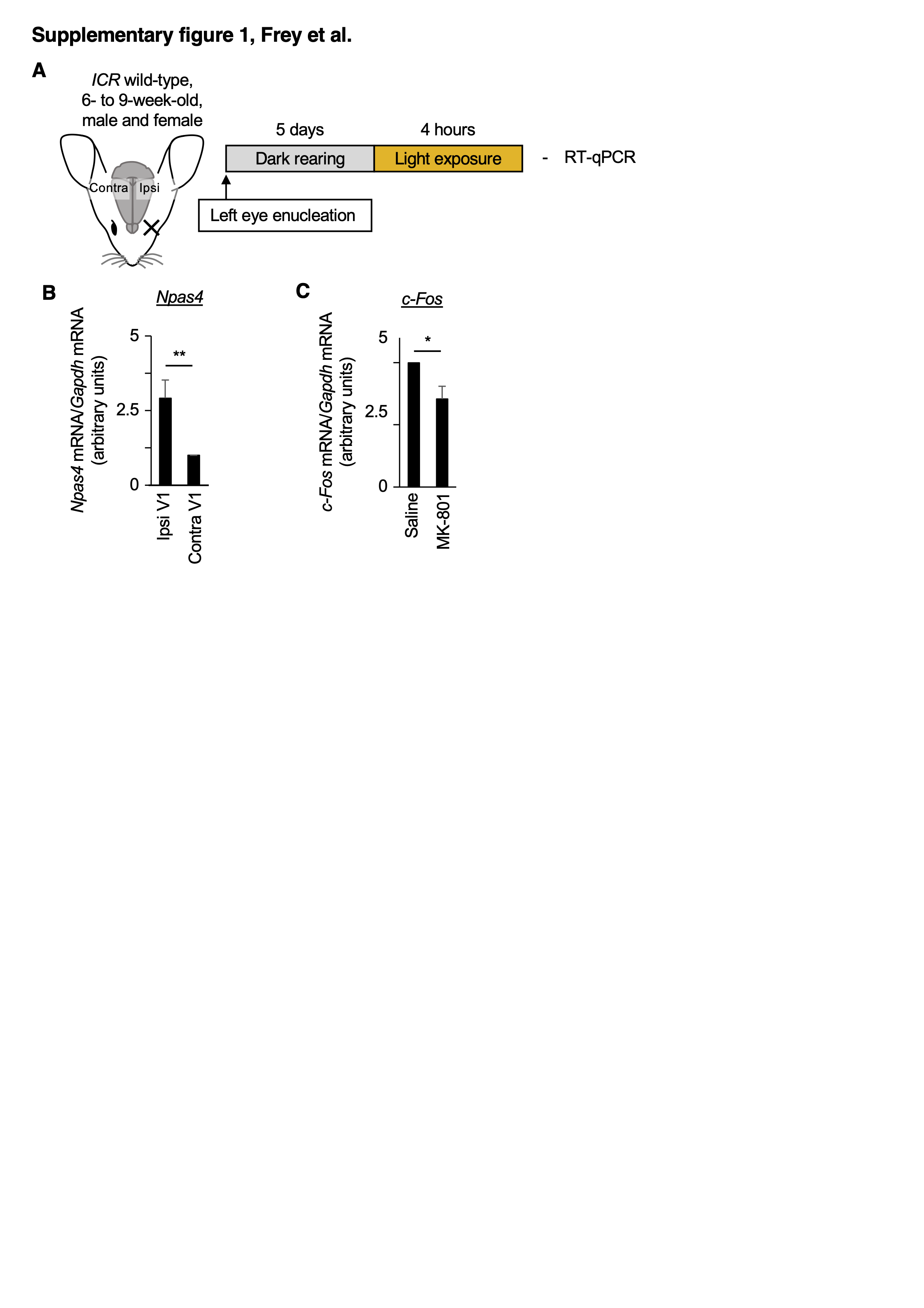

Supplement: Supplementary file 1 — Data S1. [file ACEL-22-e13925-s001.zip › acel13925-sup-0001-FigureS1.tiff]

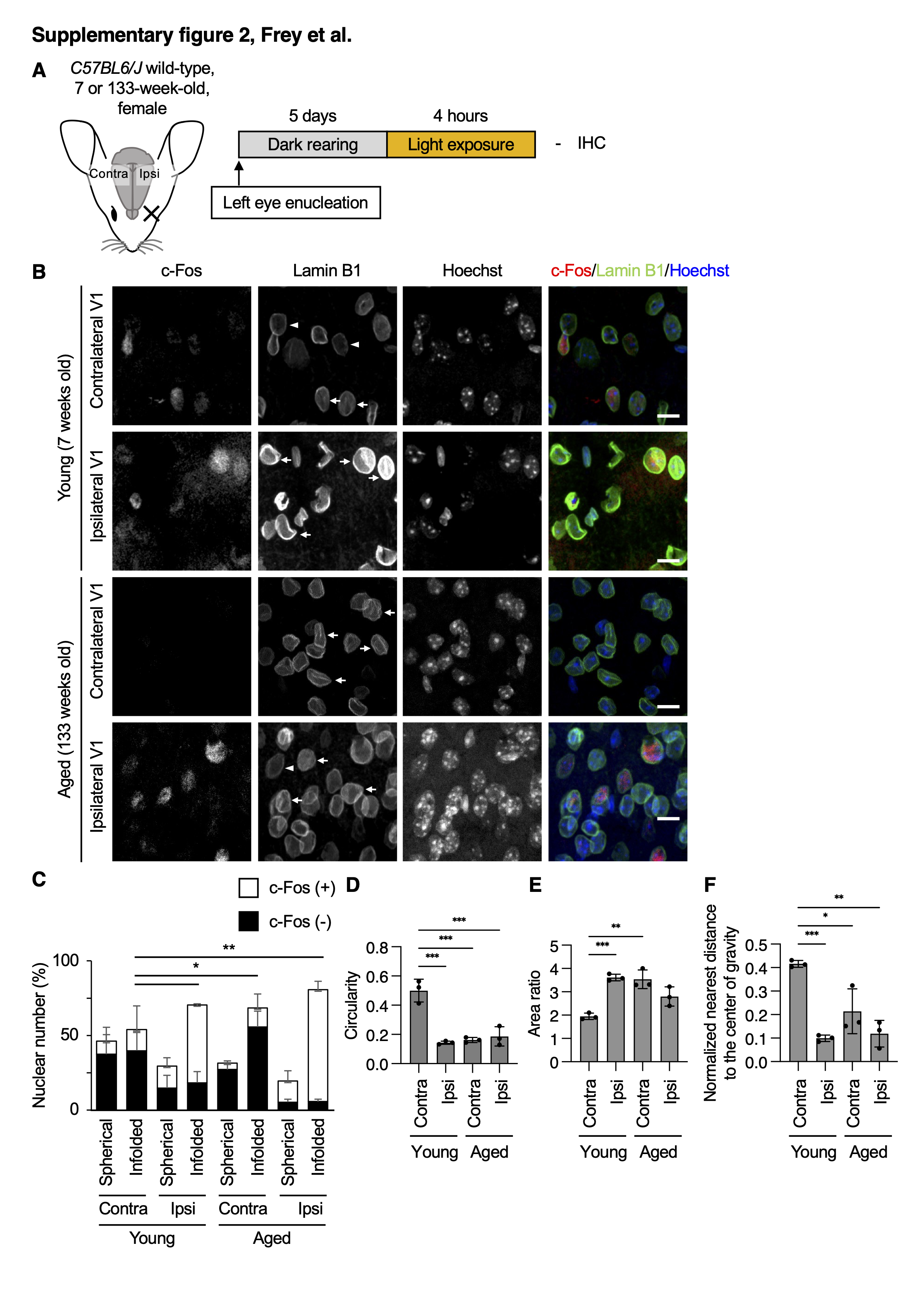

Supplement: Supplementary file 1 — Data S1. [file ACEL-22-e13925-s001.zip › acel13925-sup-0002-FigureS2.tiff]

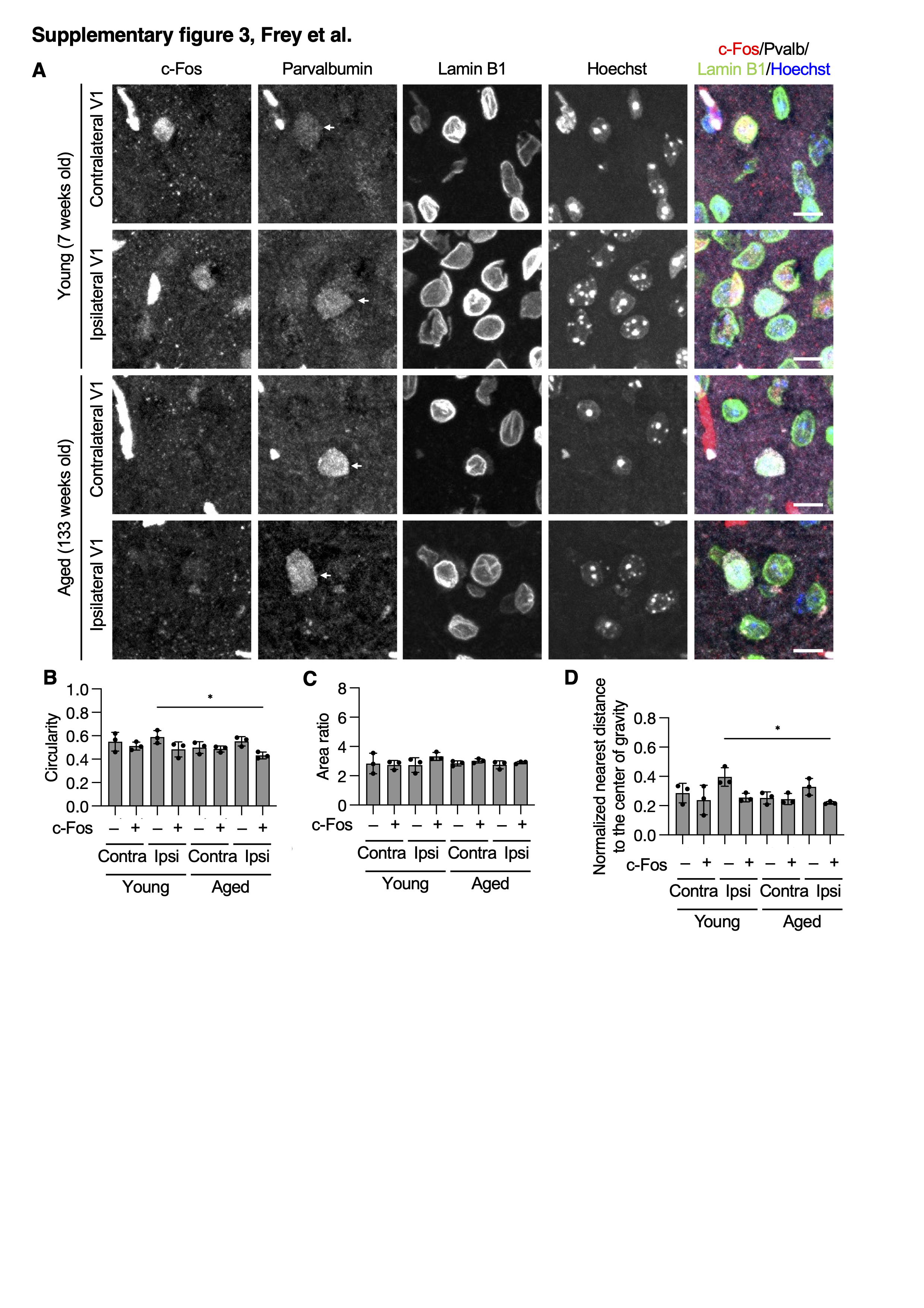

Supplement: Supplementary file 1 — Data S1. [file ACEL-22-e13925-s001.zip › acel13925-sup-0003-FigureS3.tiff]

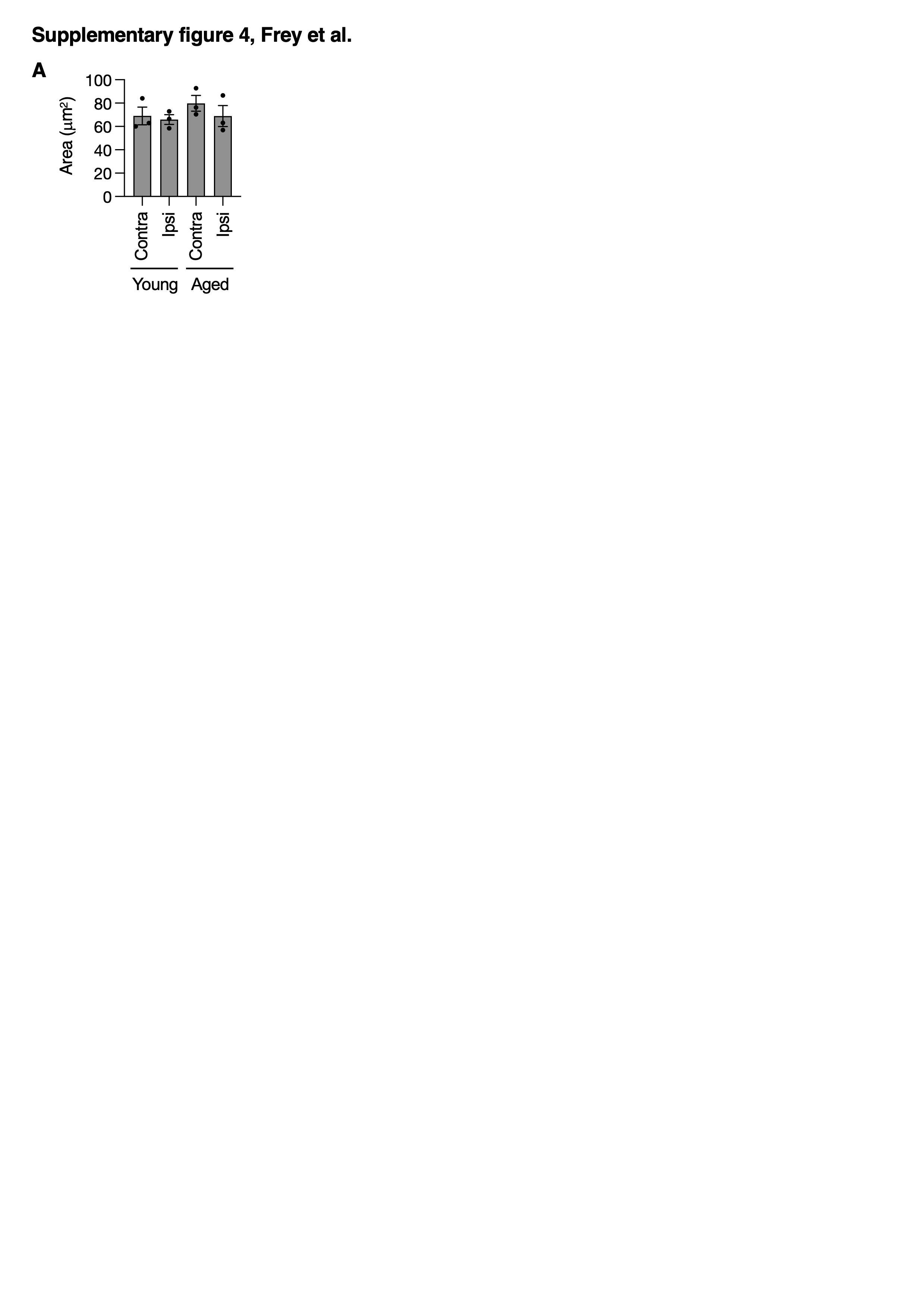

Supplement: Supplementary file 1 — Data S1. [file ACEL-22-e13925-s001.zip › acel13925-sup-0004-FigureS4.tiff]
